# Anti-angiotensin converting enzyme (ACE) proteins from mycelia of *Ganoderma lucidum* (Curtis) P. Karst

**DOI:** 10.1186/1472-6882-13-256

**Published:** 2013-10-04

**Authors:** Nurhuda Mohamad Ansor, Noorlidah Abdullah, Norhaniza Aminudin

**Affiliations:** 1 Mushroom Research Centre, Institute of Biological Sciences, Faculty of Science, University of Malaya, Kuala Lumpur 50603, Malaysia; 2 University of Malaya Centre for Proteomics Research, University of Malaya, Kuala Lumpur 50603, Malaysia; 3 Advanced Medical and Dental Institute, Universiti Sains Malaysia, Bandar Putra Bertam, Kepala Batas 13200, Penang, Malaysia

**Keywords:** ACE inhibitory proteins, Bioactive peptides/proteins, Lingzhi, Hypertension, MALDI-TOF/TOF MS

## Abstract

**Background:**

*Ganoderma lucidum* has been purported as a potent remedy in the treatment and prevention of several ailments, including hypertension. This study aimed to explore the anti-ACE potential of protein fractions from the mycelia of *G. lucidum*.

**Methods:**

*Ganoderma lucidum* mycelia were cultivated by submerged fermentation in a liquid medium containing brown sugar and spent brewer’s yeast. Intracellular proteins were fractionated from mycelia crude water extract by ammonium sulphate precipitation, and their angiotensin converting enzyme inhibitory activity was evaluated. The potential anti-ACE protein fractions were further separated by RP-HPLC and characterised using proteomics platforms.

**Results:**

Preliminary result demonstrated that the mycelia crude water extract inhibited ACE at IC_50_ value of 1.134 ± 0.036 mg/mL. Following protein fractionation and HPLC purification, the presence of highly potential anti-ACE proteins with the IC_50_ values less than 200 μg/mL was detected. Characterisation of these proteins demonstrated the presence of four different antihypertensive-related proteins involved in the regulation of blood pressure through different mechanisms.

**Conclusions:**

This study suggests that the mycelia of *G. lucidum* has high potential in lowering blood pressure level due to the presence of several antihypertensive-related proteins such as cystathionine beta synthase-like protein, DEAD/DEAH box helicase-like protein, paxillin-like protein, and alpha/beta hydrolase-like protein.

## Background

Hypertension is recognised as a grievous global health problem due to tremendous increase in the frequency of hypertensive patients as well as the catastrophic sequela caused by its occurrence. Current report from the World Health Organization [1] revealed the predominance of hypertension in approximately 40% of adults aged 25 and above worldwide. Apparently, the high prevalence has caused hypertension to emerge as one of the major contributors to cardiovascular disease (CVD).

A holistic prevention and treatment is urged in order to reduce the occurrence of the disease. In essence, practising a healthy lifestyle is a good way in preventing hypertension and would be recommended as the first-line therapy. Nevertheless, in situations where lifestyle modification alone could not adequately lower the blood pressure, antihypertensive medications will be considered.

The paramount aim in treating hypertension typically is to bring down the blood pressure to its normal level. The blood pressure in the human body is regulated by a series of enzymatic reactions in the renin angiotensin aldosterone system (RAAS). Angiotensin converting enzyme (ACE) (EC 3.4.15.1) is one of the members of RAAS, and its wide distribution throughout the body indicates that ACE plays an important role in that particular system [2]. Catalysis by ACE on angiotensin I produces angiotensin II, a potent vasoconstrictor that acts directly on vascular smooth muscle cells. Besides, the presence of angiotensin II also leads to volume expansion through sodium and fluid retention. Therefore, the inhibition of ACE has been viewed as a therapeutic target for the treatment of hypertension.

ACE inhibitors were discovered as bradykinin-poten-tiating peptides from the venom of the South American snake *Bothrops jararaca*, as well as other venomous snakes. Based on the previous findings, Cushman and Ondetti developed captopril, which became the first clinically approved synthetic ACE inhibitor [3]. There are an array of ACE inhibitor drugs available, including captopril, enalapril, fosinopril, lisinopril, perindopril, ramipril, trandolapril, and zofenopril. These ACE inhibitors differ from each other with respect to their molecular structure, potency, bioavailability, plasma half-life, and tissue affinity [4].

Consumption of synthetic ACE inhibitors was reported to bring side effects to humans. The presence of the sulphydryl binding group was suggested to attribute to skin rash, taste disturbance, and proteinuria [5]. Earlier study showed the direct effect of ACE inhibitors towards the activation of the bradykinin receptor, and the polymorphism of the receptor gene was suggested to colligate with cough [6]. Hence, the divulgence of synthetic ACE inhibitors’ side effects by clinical trials has inspired scientists to investigate a safer alternative.

Attempts have been made to explore natural products as cure for hypertension including ACE inhibitory peptides. These peptides have been increasingly acknowledged since they are less expensive and serve safer blood pressure lowering effect compared to conventional ACE inhibitor drugs. Hitherto, various ACE inhibitory peptides have been discovered in food proteins derived from dairy and marine products, fruits as well as vegetables [7]. Increased dietary intake of plant protein was reported to exert a more beneficial effect on blood pressure compared to protein from animals [8].

*Ganoderma lucidum* (Curtis) P. Karst (lingzhi, reishi) is a well-known medicinal mushroom particularly in China, Japan, and Korea. For centuries, the fruiting bodies of *G. lucidum* have been adopted by old folks to treat various ailments. However, the production of the fruiting bodies is time-consuming because the cultivation of basidiocarp requires at least three to five months. The production of *G. lucidum* mycelia used as a bioresource of peptides is approximately six times faster than when fruiting bodies are utilised. Therefore, it leads to the preference of mycelia as an alternative panacea.

Recent research reports create awareness on the health-improving properties of the mycelia [9] and thus urge the need for more comprehensive studies. To date, no study has been conducted to determine if the mycelia proteins exhibit anti-ACE properties. Hence, the present study aims to isolate and characterise the peptides/proteins with promising anti-ACE activity from the mycelia of *G. lucidum*.

## Methods

### Maintenance and cultivation of *Ganoderma lucidum* mycelia

*Ganoderma lucidum* (Curtis) P. Karst culture (KUM50079) was kindly provided and authenticated by the Mushroom Research Centre, University of Malaya. It was maintained on malt extract agar (Oxoid Ltd.) slants at 25°C. During submerged cultivation, the mycelia were grown in a liquid medium consisting of 2% (w/v) brown sugar and 1% (w/v) spent brewer’s yeast at pH 5. The cultivation was performed in 500 mL Erlenmeyer flasks, each containing 100 mL of media. Ten mycelia plugs (10 mm diameter) were cut from the periphery of a 12-day-old culture using a sterile cork borer and were inoculated into each sterilised flask. The flasks were incubated at room temperature and agitated at 140 rpm using SK 300 rotary shaker (Lab Companion). Following seven days of cultivation, the mycelia were harvested. Mycelia biomass was separated from the broth culture by employing vacuum filtration (Jeio Tech, Korea) and washed with a large amount of distilled water. Later on, the mycelia biomass was freeze-dried (Labconco), and the dried crude extract was preserved at 4°C.

### Preparation of crude mycelial extract

Freeze-dried mycelia (10 mg) were crushed using mortar and pestle. The crushed sample was dissolved in 200 mL of distilled water (ratio 1: 20). Extraction was carried out at a low temperature of 4°C with constant stirring. Subsequently, the water extract was subjected to centrifugation at 4°C and 5000 rpm for 20 minutes. The pellet formed was discarded, while the supernatant was kept for further study.

### Fractionation of mycelial proteins by ammonium sulphate precipitation

Partial purification was carried out by employing ammonium sulphate precipitation method. Salt (11.2 g) was slowly added to mycelia crude water extract (200 mL) with gentle stirring on an ice bath. The solution was centrifuged at 10,000 rpm (4°C) for 15 minutes. The solution was then subjected to gradual increase of salt (20% to 100%). Pellet obtained was redissolved in a minimum amount of distilled water and later dialysed using SnakeSkin pleated dialysis tubing with 3,500 Da molecular weight cutoff (Thermo Scientific). The process was performed at 4°C for 48 hours with four times buffer changes. Afterwards, the dialysed proteins were freeze-dried and stored at -20°C.

### Grouping of fractionated proteins based on sodium dodecyl sulphate-polyacrylamide gel electrophoresis (SDS-PAGE) profile

SDS-PAGE analysis for dialysed mycelial proteins (10% to 100%) was performed based on the discontinuous buffering system. The protein separation was done on 18% polyacrylamide Tris/HCl gels. The analysis was carried out under reducing condition with the addition of dithiothreitol (5 μL, 1% (w/v)) to the protein solutions (15 μL, 1 μg/μL). All samples were heated at 90°C for 5 minutes prior to electrophoresis. Prestained SDS-PAGE standards broad range molecular weight (Bio-Rad) was used as the molecular markers. The gel was stained with silver staining to visualise the protein bands. Dialysed proteins (10% to 100%) showing similar protein band profiles were pooled together as fractions and subjected to ACE inhibitory activity evaluation.

### Evaluation of ACE inhibitory activity of mycelial proteins

The ACE inhibitory activity of the protein fractions was evaluated using ACE Kit-WST (Dojindo, Japan). The inhibitory activity was measured by the detection of 3-hydroxybutyric acid at 450 nm and involved a series of enzyme reactions. Protein concentration was determined using Pierce BCA Protein Assay Kit (Thermo Fisher Scientific) and observed spectrophotometrically at 562 nm. Bovine serum albumin was utilised as the standard reference.

### Reversed phase HPLC purification of active ACE inhibitory proteins

Protein fractions having the strongest inhibitory activity were subjected to reversed phase HPLC (RP-HPLC) (Shimadzu, Japan). The purification was carried out on an Atlantis® T3 C_18_ column; 5 μm particle size, 250 mm × 4.6 mm (Waters Corporation, Ireland). Fraction A was purified using elution buffer 5% to 90% acetonitrile for 45 minutes with the flow rate 1.0 mLmin^-1^. Fraction C used elution buffer 5% to 90% acetonitrile for 40 minutes with the flow rate 1.0 mLmin^-1^. Eluted samples were monitored using a PDA detector at 220 nm and 254 nm. Individual HPLC peaks were collected, and acetonitrile was totally removed under a stream of nitrogen gas. Later, the samples were freeze-dried and evaluated for anti-ACE activity as described earlier.

### Identification of protein components in active HPLC eluted peaks by SDS-PAGE and MALDI TOF/TOF MS

HPLC peaks with potent ACE inhibitory activity were further separated using SDS-PAGE. Protein bands from SDS-PAGE gel were excised and subjected to in-gel tryptic digestion (6 ng/μL, 37°C overnight). Digested samples were then desalted using Zip Tip (C18) (Millipore) and analysed using 4800 Plus MALDI TOF/TOF MS (Applied Biosystems) [10] combined with Mascot database software (available at http://www.matrixscience.com). Unmatched masses from the Mascot database search were further analysed by an alternative protein identification tool, ProFound (available at http://prowl.rockefeller.edu/prowl-cgi/profound.exe) using the following parameters: taxonomy category - fungi, protein mass range - 0 kDa to 100 kDa, protein pI range - 3 to 10, one missed cleavage site, charge state of MH+, mass tolerance between 0.5 Da to 1.15 Da.

### Statistical analysis

Data analysis was performed using Minitab statistical software (Minitab Incorporation, USA). The effect of ACE inhibitor on the activity of ACE was tested by one-way ANOVA. Means were accepted as significantly different at 95% confidence level (*P* < 0.05).

## Results and discussion

In the present study, preliminary evaluation revealed that the mycelia crude water extract actively inhibited ACE activity with the IC_50_ value of 1.134 ± 0.036 mg/mL compared to broth extract (data not shown). IC_50_ was defined as the concentration of sample that inhibited 50% of ACE activity under the experimental conditions. The ACE inhibitory potential of *G. lucidum* mycelia was found to be approximately 1.82-fold stronger when compared to *Lyophyllum decastes* mycelia (IC_50_ = 1.637 ± 0.057 mg/mL) [11]. The results revealed the importance of mycelia as the source of ACE inhibitors that can be obtained by liquid fermentation.

In order to track down the responsible proteins for the inhibitory activity, the crude water extract was subjected to protein purification, first, by using salting-out method. Based on the salt saturation, ten mycelial proteins were obtained (10% to 100%). Upon visualisation of SDS-PAGE gel, fractions that shared similar protein band profiles were pooled and grouped together, resulting in five fractions: fraction A (10%-40% salt saturation), fraction B (50%-60% salt saturation), fraction C (70% salt saturation), fraction D (80%-90% salt saturation) and fraction E (100% salt saturation).

**Figure 1:**
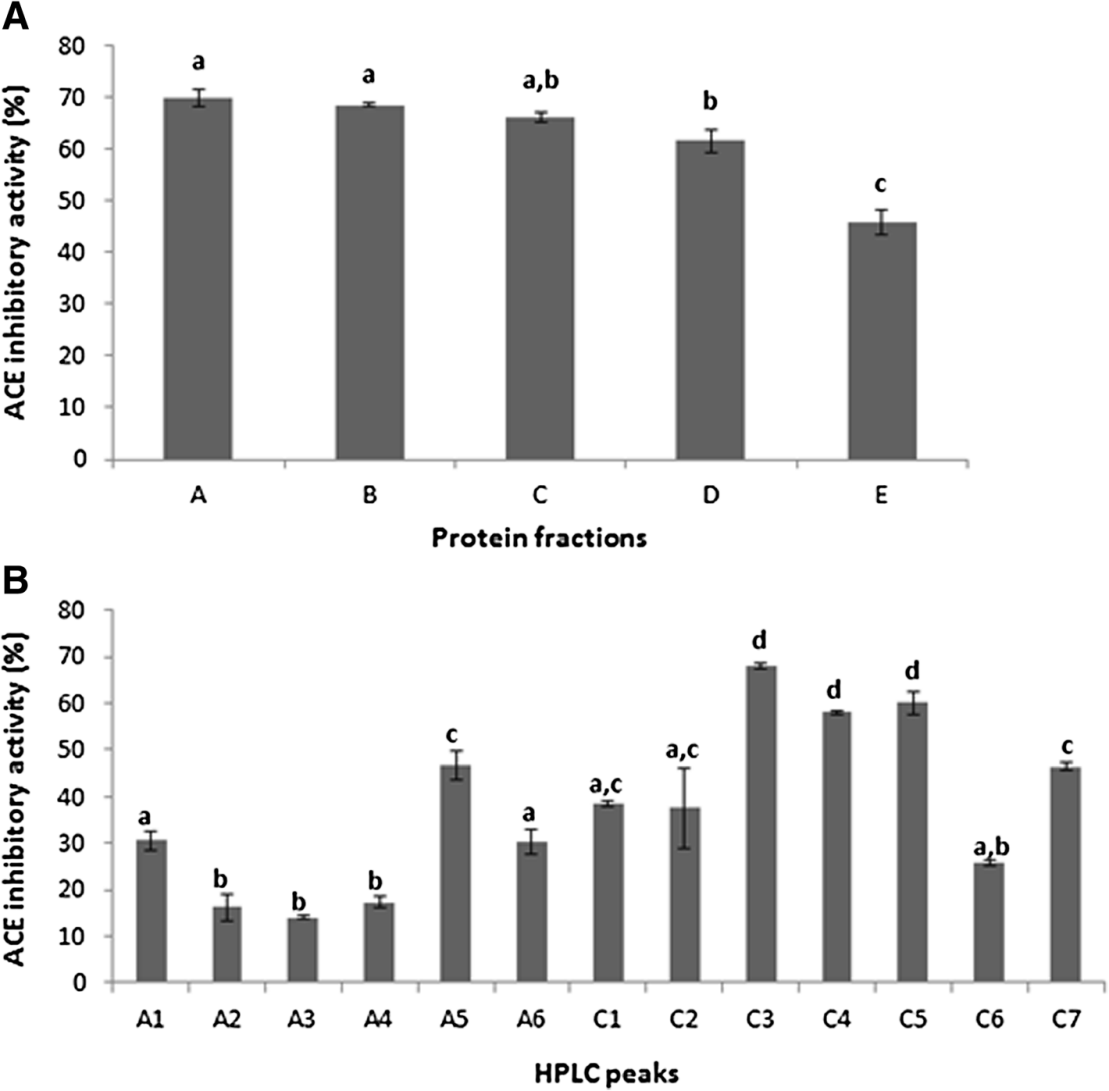
**ACE inhibitory activity of (A) protein fractions at a concentration of 200 μg/mL and (B) RP-HPLC peaks evaluated at a concentration of 25 μg/mL.** Values are expressed as mean ± SEM of three replicate determinations. Mean values with different lowercase letters (a**-**d) indicate significant difference at P < 0.05.

ACE inhibitory activity was measured for all fractions at a concentration of 200 μg/mL (Figure 1A). Fractions A (69.92% ± 2.71%, IC_50_ = 120 ± 2.65 μg/mL), B (68.54% ± 0.49%, IC_50_ = 125 ± 4.62 μg/mL), and C (66.08% ± 1.71%, IC_50_ = 109 ± 1.53 μg/mL) showed more than 65% inhibition percentage. Since fraction C demonstrated the strongest inhibition and gave the lowest IC_50_ value among all, it was selected to be further purified. Fraction A was also considered for further protein purification step. This was due to its properties of being the more hydrophobic than others and concurrently showing potent inhibitory activity. Polarity of inhibitor seemed to have a notable effect on ACE inhibition as the presence of inhibitor possessing abundant hydrophobic amino acid residues was suggested to contribute to high ACE inhibitory activity [12].

**Figure 2:**
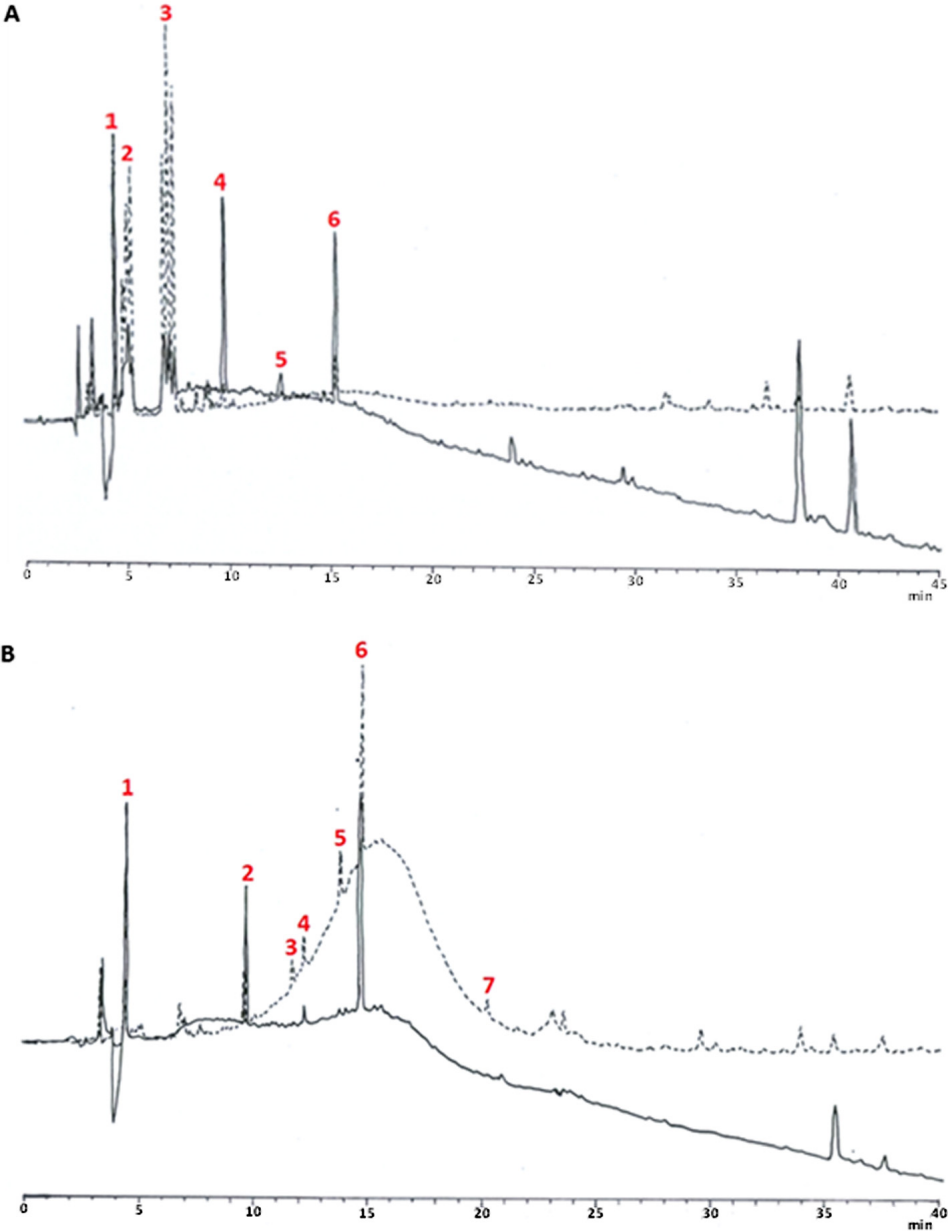
**RP-HPLC profile of (A) protein fraction A and (B) protein fraction C.** Numbers 1**-**7 indicate the peaks that were collected for further analysis. Indicates wavelength at 220 nm;indicates wavelength at 254 nm.

Both fractions A and C were further separated by RP-HPLC on a C_18_ column. Figure 2 represents the superimposed HPLC profiles of separated proteins acquired at wavelengths 220 nm and 254 nm. Eluted peaks derived from protein fractions A (Figure 2A) and C (Figure 2B) are tagged as A1**-**A6 and C1**-**C7, respectively. The evaluation of ACE inhibitory activity at 25 μg/mL samples demonstrated that only C3 (67.94% ± 1.04%), C4 (57.96% ± 0.64%), and C5 (60.06% ± 3.37%) showed more than 50% inhibition (Figure 1B).

The IC_50_ values determined for these peaks were 10 ± 0.15, 18 ± 1.62, and 12.5 ± 1.71 μg/mL, respectively (Table 1). The results indicated that separated proteins exhibited stronger inhibitory activity than a whole protein. The inhibitory effect was stronger compared to the naturally occurring ACE inhibitors discovered from other medicinal mushrooms such as *Grifola frondosa* (IC_50_ = 97 μg/mL), *Tricholoma giganteum* (IC_50_ = 40 μg/mL), and *Pholiota adiposa* (IC_50_ = 44 μg/mL) [13-15].

**Table 1:** **IC**_**50 **_**of ACE inhibitory proteins from *G. lucidum *mycelia**

**Protein source**	**IC**_**50 **_**values (μg/mL)**^**a**^
Crude water extract	1134 ± 36
*Ammonium sulphate precipitation*	
Fraction A	120 ± 2.65
Fraction C	109 ± 1.53
*RP-HPLC*	
C3	10.0 ± 0.15
C4	18.0 ± 1.62
C5	12.5 ± 1.71

^a^Data are expressed as mean ± SD of three replicate determinations for each purification step.

Conversely, peaks from fraction A demonstrated meagre inhibitory rate. This result could suggest synergistic interaction between proteins in fraction A since they effectively inhibited ACE activity as a group but the inhibition effect became insignificant once they were separated through chromatography. Apparently, the synergistic effect of ACE inhibitory peptides had been reported by other studies [16].

**Figure 3:**
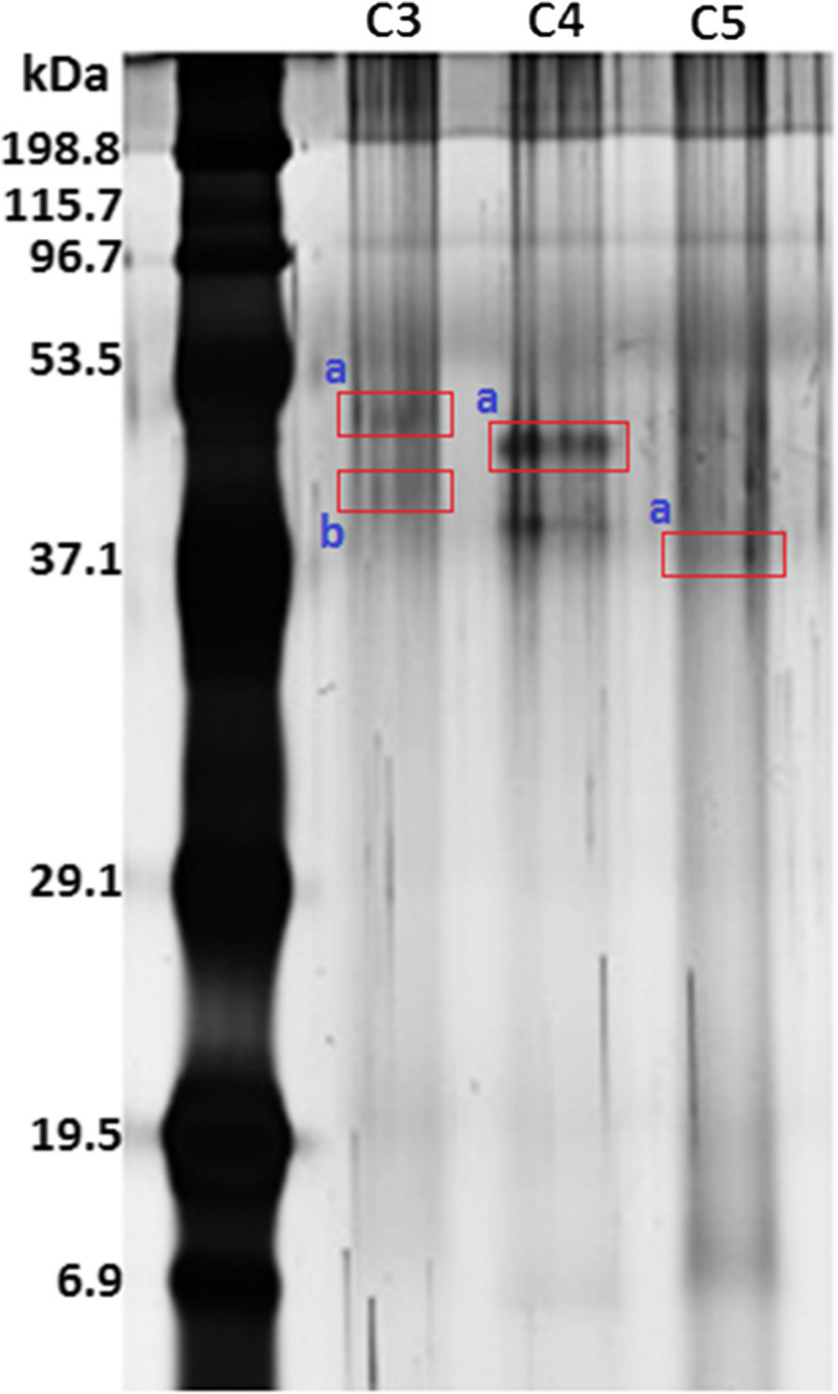
**SDS-PAGE profile for HPLC peaks C3, C4, and C5.** Identified antihypertensive-related proteins are marked in boxes with alphabetical a**-**b.

Protein profiling of the potent anti-ACE HPLC peaks (C3-C5) was performed by SDS-PAGE (Figure 3), and distinct protein bands observed were subjected to protein identification using a proteomic tool, MALDI TOF/TOF MS. In MALDI, the protein samples were digested by trypsin, and the masses of all peptides generated were shown in MS spectrum. The search for matched protein was later done by using peptide mass fingerprinting (PMF) method in which peptides’ masses attained from MALDI were incorporated as the fingerprint in Mascot database. It appeared that the score for every sample spot was low and not significant at the 0.05 level of confidence, and thus revealed that the unknown proteins were less identical to the proteins in the database of the Mascot program.

An alternative PMF program, ProFound, had been adopted to identify the unknown proteins since the results provided by Mascot were less favourable. ProFound was selected after considering its good performance in identifying proteins [17]. The peptides’ masses obtained from MALDI TOF/TOF MS were submitted to the ProFound program, and intriguingly, four antihypertensive-related proteins were recognised as the best matched protein candidate (Table 2). Each of the identified proteins has a probability of approximately 1 and this value referred to the value calculated according to Bayesian probability. It was suggested that protein candidate having a probability value close to 1 was likely to be the correct protein [18].

**Table 2:** ProFound search results for unknown proteins

			**Sequence**	**Estimated**
**Putative**	**Probability**	**Estimated**	**coverage**	**molecular**
**protein**		**Z score**^**b**^		
			**(%)**^**c**^	**weight (kDa)**
Cystathionine beta synthase	1.00e + 000	2.43	29	49.0
DEAD/DEAH box helicase	1.00e + 000	2.43	19	43.7
Paxillin	1.00e + 000	2.43	23	46.8
Alpha/beta hydrolase	1.00e + 000	2.43	18	37.1

^a^Value is calculated according to Bayesian probability.

^b^The score reflects the quality of the search result in which it indicates measure of the confidence for ProFound database. Z score of 2.43 correlated to a false-positive rate of 1%.

^c^Sequence coverage is defined as the ratio of the length of the protein sequence covered by the matched peptides to the whole protein sequence.

In addition, the quality of the search result was also reported in which it was represented in the form of an estimated Z score. Z scores of more than 1.645 were considered significant (*P* < 0.05). Each of the putative proteins was found to have a high Z score, which was 2.43, indicating the respective search result having 99% probability of an accurate match. Apparently, it revealed that unambiguous protein identification was achieved using the ProFound tool. These proteins were involved in the regulation of blood pressure through various mechanisms. Hence, the unknown proteins were characterised as cystathionine beta synthase-like protein (C3-a), DEAD/DEAH box helicase-like protein (C3-b), paxillin-like protein (C4-a), and alpha/beta hydrolase-like protein (C5-a) (Figure 3).

Cystathionine beta synthase (CBS) is involved in the transsulfuration pathway and is crucial for the production of hydrogen sulfide (H_2_S). CBS might also be involved in blood pressure control, in which it is assumed to inhibit ACE activity by interfering with Zn^2+^ in the active centre of ACE. Findings from Proudfoot and colleagues showed that CBS has been recognised to possess metal ions binding properties due to the presence of the Zn ribbon domain [19]. In an earlier study, this Zn ribbon domain was suggested to possess the ability to bind zinc [20]. Zinc cofactor is a prominent component for the enzyme catalysis, and therefore, the interaction of inhibitor with Zn^2+^ at the active centre could disrupt ACE activity. Inactivation of ACE resulted in the decreased production of vasoconstrictor substance, angiotensin II.

With regard to the present results, we assumed that the matched homology of CBS-like protein to that of CBS might include protein sequences of the Zn ribbon domain. Further work is required to validate whether CBS-like protein inhibits ACE by interacting with Zn^2+^ at the enzyme’s active centre. A study on structure-activity relationship is a good approach to justify the postulation [21].

The second related antihypertensive protein belongs to the DEAD/DEAH box helicase family. DEAD/DEAH box helicase is commonly involved in cellular activities. Recently, DEAD/DEAH box helicase was suggested to exhibit antihypertensive effect through the regulation of cardiac cellular activities instead of RAAS [22]. According to Liu and Olson, cardiac helicase activated by MEF2 protein (CHAMP), which belongs to DEAD box proteins, had shown to suppress the growth of cardiomyocyte in cardiac hypertrophy [23]. CHAMP demonstrated antihypertrophic activity by inhibiting proliferation through general cell cycle machinery and was characterised by the up regulation of the cell cycle inhibitor p21^CIP1^. The inhibitory activity prompted the regression of cardiac hypertrophy and thus recovered diastolic function and coronary flow reserve [24]. The recovery of the blood pressure and flow advantageously contributed to blood pressure lowering effect.

Based on our result, DEAD/DEAH box helicase-like protein might be the responsible protein for the ACE inhibitory activity, and at the same time, it might also demonstrate hypotensive effect via cellular mechanism. This synergistic effect may bring considerable success in treating hypertension in the future. A caution, of course, is that the thorough mechanism of DEAD/DEAH box helicase-like protein is beyond our meagre comprehension. Thus, a more detailed investigation on the structural and pharmacological properties is suggested to be carried out.

The third antihypertensive-related protein is paxillin. In general, paxillin serves primary functions in the signalling pathway and cell motility due to the presence of multidomain bindings [25]. Since paxillin lacks identifiable enzymatic activity, it was suggested that paxillin plays a major role as an adaptor molecule that interacts with regulatory proteins. Intriguingly, paxillin has been found to associate with the lowering blood pressure ability through the regulation of the vascular smooth muscle.

Positioned at the dense plaques of the smooth muscle tissue, paxillin acts as the signalling protein mediated actin cytoskeleton remodelling process [26]. Polymerisation and depolymerisation of the actin filaments resulted in the contraction and relaxation of the vascular smooth muscle. The vasodilation of the vascular smooth muscle has been understood to trigger blood pressure reduction in hypertensive patients. Thus, paxillin mediated the action of actin cytoskeleton on blood pressure by promoting the relaxation of the smooth muscle [27].

Paxillin-like protein might be the significant protein that contributed to the potent anti-ACE activity. While exhibiting partial homology with paxillin, we assumed that paxillin-like protein might induce the vasodilation of the vascular smooth muscle. It is anticipated that this action will be additive to the ACE inhibitory activity of paxillin-like protein and may possibly provide a beneficial response beyond antihypertension treatments described to date. Further studies are needed to resolve these and other possibilities.

The fourth antihypertensive-related protein is alpha/beta hydrolase. Current study has discovered the contribution of alpha/beta hydrolase protein in blood pressure regulation through the involvement of soluble epoxide hydrolase (sEH) [28]. Their data was in agreement with the findings from Yu and coworkers in which antihypertensive effect was observed following sEH inhibition [29].

Apparently, inhibition on sEH activity demonstrated a reduction in blood pressure and has been the target for treating hypertension. Inhibitory activity on sEH resulted in increased epoxyeicosatrienoic acids (EET) level and decreased dihydroxyeicosatrienoic acids (DHET) level. Accordingly, blood pressure lowering event could be explained by the increased release of EET that potentiated a vasorelaxant response [30]. EET acts as an endothelium-derived hyperpolarising factor where it induced the vasodilation of the vascular smooth muscle.

In our study, alpha/beta hydrolase-like protein was expected to exert anti-ACE properties, and the detailed mechanism of the inhibition still remained unclear. It was speculated that alpha/beta hydrolase-like protein might also restore blood pressure level by acting as an alpha/beta hydrolase inhibitor. On the account that sEH inhibition could reduce inflammation, it is anticipated that alpha/beta hydrolase-like protein might provide synergistic effects in hypertensive patients. The understanding of alpha/beta hydrolase-like protein action is far from complete, and it is critical to perform an in-depth investigation.

## Conclusions

*Ganoderma lucidum* has drawn folk’s attention in improving health status in which it could ameliorate ailments and lessen diseases risk. In this study, we have revealed the ability of *G. lucidum* mycelia in lowering blood pressure level through the inhibition of ACE activity. Following purification and identification, we have discovered four promising antihypertensive-related proteins which are cystathionine beta synthase-like protein, DEAD/DEAH box helicase-like protein, paxillin-like protein, and alpha/beta hydrolase-like protein. These proteins (derived from edible mushrooms) are suggested to have a lesser possibility to exert adverse side effects and hence can be a good alternative to conventional antihypertensive drug treatment. This seems to have a remarkable positive impact on public health across the globe. All in all, this work had emphasised on *in vitro* ACE inhibitory activity, HPLC purification, and identification of the potential antihypertensive-related proteins by proteomics platforms. Further isolation for pure ACE inhibitor, elucidation of the relationship between ACE inhibitor’s structure and activity, and *in vivo* studies are warranted to ensure its application as a safe alternative to antihypertensive treatment for humans.

## Abbreviations

CVD: Cardiovascular disease; RAAS: Renin angiotensin aldosterone system; ACE: Angiotensin converting enzyme; EC: Enzyme class; G. lucidum: *Ganoderma lucidum*; SDS-PAGE: Sodium dodecyl sulphate-polyacrylamide gel electrophoresis; RP-HPLC: Reversed phase HPLC; IC50: Medium inhibitory concentration; PMF: Peptide mass fingerprinting; CBS: Cystathionine beta synthase; H2S: Hydrogen sulfide; CHAMP: Cardiac helicase activated by MEF2 protein; sEH: Soluble epoxide hydrolase; EET: Epoxyeicosatrienoic acids; DHET: Dihydroxyeicosatrienoic acids.

## Competing interests

The authors declare that they have no competing interests.

## Authors’ contributions

NMA carried out all the laboratory works. NA and NA participated in designing as well as in coordinating the study. All authors contributed to the manuscript preparation, read, and proofed the final manuscript.

## Pre-publication history

The pre-publication history for this paper can be accessed here:

http://www.biomedcentral.com/1472-6882/13/256/prepub
